# Supplementary material for: Atomically dispersed Cu coordinated Rh metallene arrays for simultaneously electrochemical aniline synthesis and biomass upgrading
Source: Nat Commun. 2023 Sep 14;14:5679. doi: 10.1038/s41467-023-41423-2 (PMC10502102; doi:10.1038/s41467-023-41423-2)
Supplement: Supplementary file 1 — Supplementary Information [file 41467_2023_41423_MOESM1_ESM.pdf]

Supplementary Information for

Atomically dispersed Cu coordinated Rh metallene arrays for simultaneously electrochemical aniline synthesis and biomass upgrading

Qiqi Mao[1], Xu Mu[1], Wenxin Wang[1], Kai Deng[1], Hongjie Yu[1], Ziqiang Wang[1], You Xu[1], Liang Wang[1] and Hongjing Wang[1]*

[1.] State Key Laboratory Breeding Base of Green-Chemical Synthesis Technology, College of Chemical Engineering, Zhejiang University of Technology, Hangzhou 310014, P. R. China.

*****Corresponding author

E-mail: hjw@zjut.edu.cn

Supplementary method

DFT calculations: Based on experimental results, the Cu_{SA}-Rh MAs display the single-phase *fcc* crystal structure and single-atom alloy structure. In the DFT simulation process, the Rh model is firstly constructed, and the Cu single-atom is induced with a random distribution, we used the *fcc* cubic phase of the original Rh as a template to build a 3×3×3 supercell containing 108 atoms for Cu_{SA}-Rh MAs, the atom ratio of Rh : Cu is approximately 93 : 7. The present first principle DFT calculations are performed by Vienna Ab initio Simulation Package (VASP) with the projector augmented wave (PAW) method[1,2]. Structural optimization calculations were performed by VASP with the PAW method. The exchange-functional is treated using the generalized gradient approximation (GGA) of Perdew-Burke-Emzerhof (PBE) functional[3]. The energy cutoff for the plane wave basis expansion was set to 400 eV and the force on each atom less than 0.05 eV/Å was set for convergence criterion of geometry relaxation. Grimme's DFT-D3 methodology[4]. was used to describe the dispersion interactions. Partial occupancies of the Kohn−Sham orbitals were allowed using the Gaussian smearing method and a width of 0.05 eV. The Brillourin zone was sampled with Monkhorst mesh 3 × 3 × 1 through all the computational process. The self-consistent calculations apply a convergence energy threshold of 10^{-5} eV. A 15 Å vacuum space along the z direction was added to avoid the interaction between the two neighboring images. The free energy changes (ΔG) of each elementary reaction step during Ph-NO_2 ERR/MOR were calculated using the computational hydrogen electrode (CHE) model. In this model, the chemical potential is equal to the energy of half of the gas-phase H_2 at 0 V vs reversible hydrogen electrode (RHE). The electrode potential, U verse RHE, is taken into consideration by adding -eU when an electron transforming step occurs. That is G (U) = G (0 V) - neU, where e is the elementary charge of an electron, n is the

number of proton-electron pairs transferred, and U is the applied potential. The Gibbs free energy was calculated by the following Supplementary equation (1):

$$\Delta G = \Delta E + \Delta E_{ZPE} - T\Delta S \quad (1)$$

Where the value of ΔE, ΔE_{ZPE} and ΔS denotes the changes of DFT energy, the zero-point energy and the entropy at 298.15 K, respective.

The adsorption energy (Eads) of adsorbate was defined as Supplementary equation (2):

$$E_{ads} = E_{Total} - E_{surf} - E_{adsorbate} \quad (2)$$

where E_{total}, E_{surf} and $E_{adsorbate}$ are the energy of adsorbate adsorbed on the surface, the energy of clean surface, and the energy of isolated molecule in a cubic periodic box, respectively.

A climbing image nudged elastic band (CI-NEB) method was used to locate the transition states with the same convergence standard.

Supplementary Figures

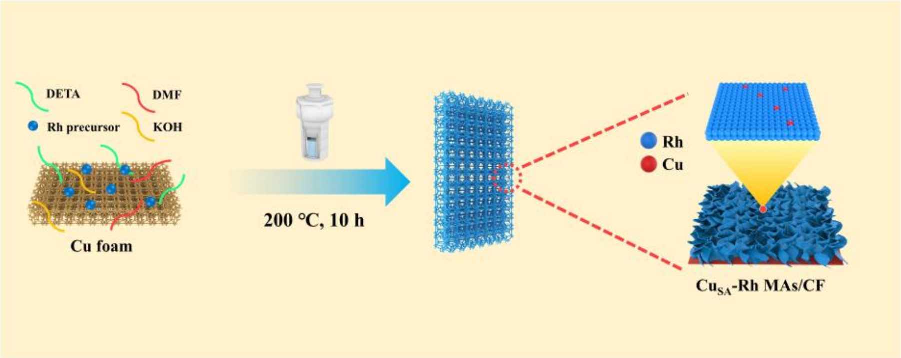

Supplementary Fig. 1. Synthesis diagram. Schematic diagram of the synthesis process for formed Cu$_{SA}$-Rh MAs/CF.

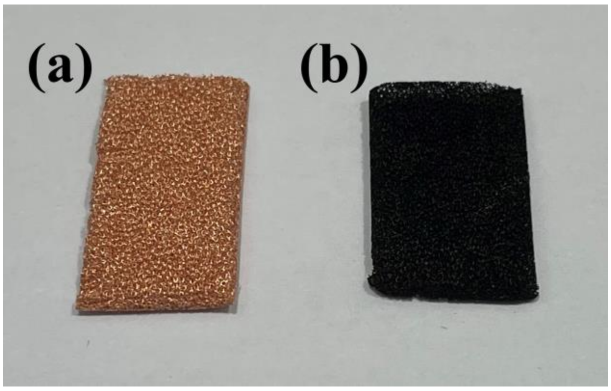

Supplementary Fig. 2. Digital photo of samples. The digital photo of (a) CF and (b) Cu$_{SA}$-Rh MAs/CF**.**

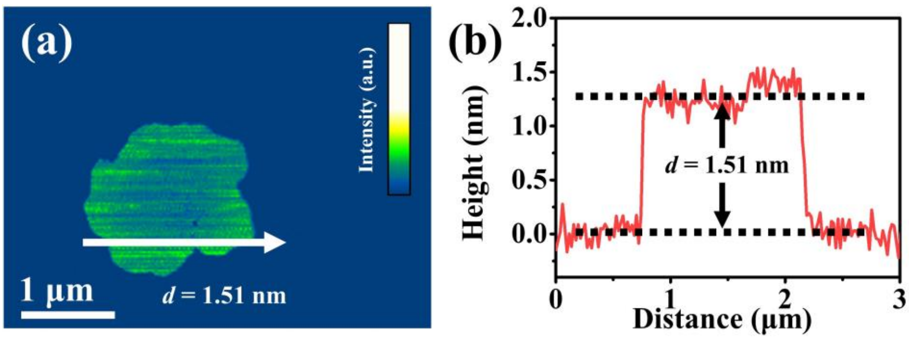

Supplementary Fig. 3. AFM characterization of Cu$_{SA}$-Rh MAs. (a) AFM image and (b) corresponding height profile of Cu$_{SA}$-Rh MAs.

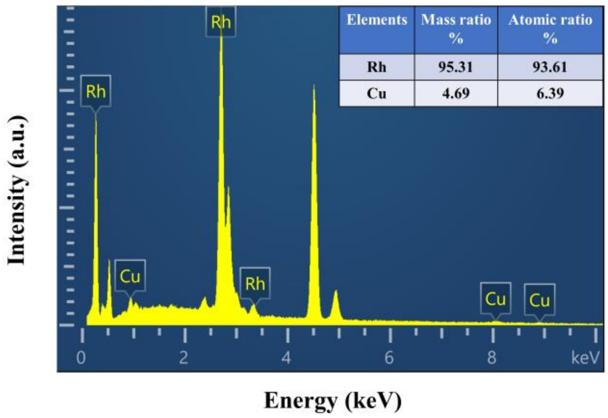

Supplementary Fig. 4. EDX spectrum analysis. EDX spectrum of Cu_{SA}-Rh MAs.

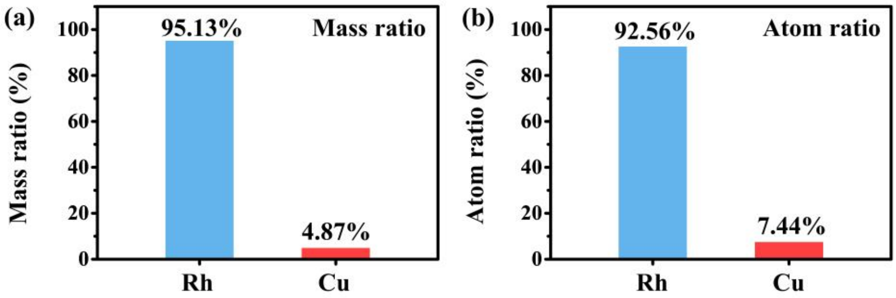

Supplementary Fig. 5. ICP-OES tests. (a) Mass ratio and (b) atom ratio of Rh and Cu elements in Cu_{SA}-Rh MAs obtained from ICP-OES.

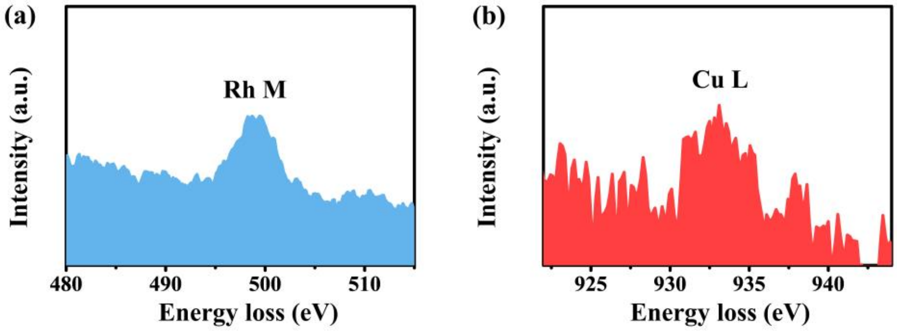

Supplementary Fig. 6. EELS tests. (a and b) The EELS spectra of Rh and Cu in Cu_{SA}-Rh MAs.

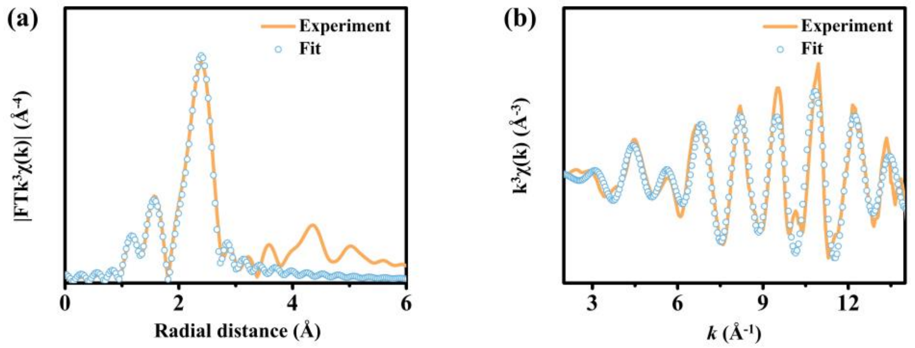

Supplementary Fig. 7. EXAFS spectra of Cu_{SA}-Rh MAs. Rh *K*-edge experimental and fitting Fourier transformed EXAFS spectra of Cu_{SA}-Rh MAs at (a) *R* space and (b) *K* space.

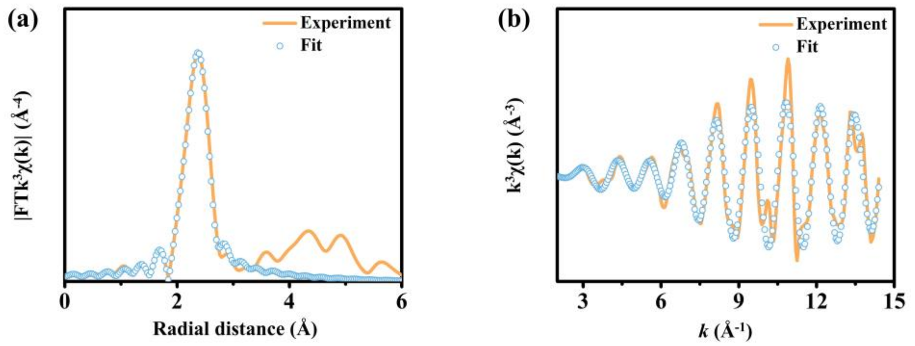

Supplementary Fig. 8. EXAFS spectra of Rh foil. Rh *K*-edge experimental and fitting Fourier transformed EXAFS spectra of Rh foil at (a) *R* space and (b) *K* space.

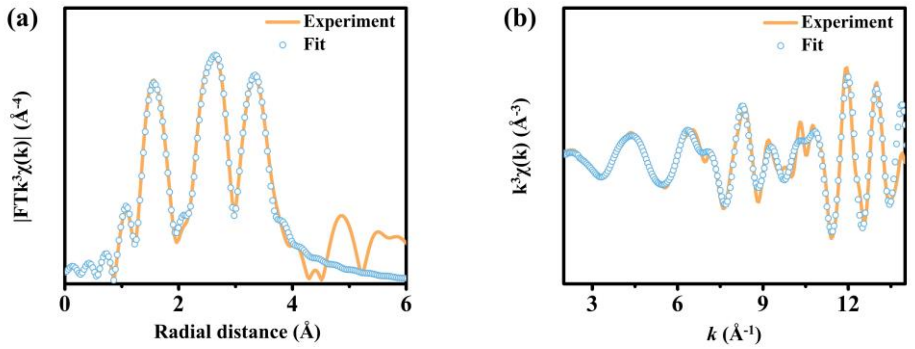

Supplementary Fig. 9. EXAFS spectra of Rh_2O_3. Rh *K*-edge experimental and fitting Fourier transformed EXAFS spectra of Rh_2O_3 at (a) *R* space and (b) *K* space.

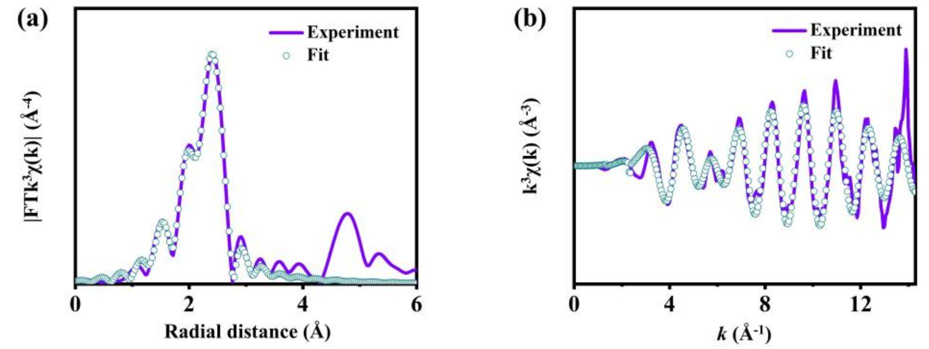

Supplementary Fig. 10. EXAFS spectra of Cu$_{SA}$-Rh MAs. Cu *K*-edge experimental and fitting Fourier transformed EXAFS spectra of Cu$_{SA}$-Rh MAs at (a) *R* space and (b) *K* space.

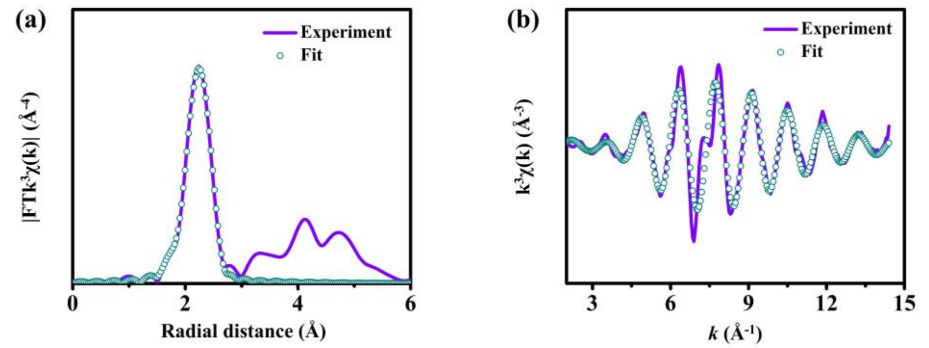

Supplementary Fig. 11. EXAFS spectra of Cu foil. Cu *K*-edge experimental and fitting Fourier transformed EXAFS spectra of Cu foil at (a) *R* space and (b) *K* space.

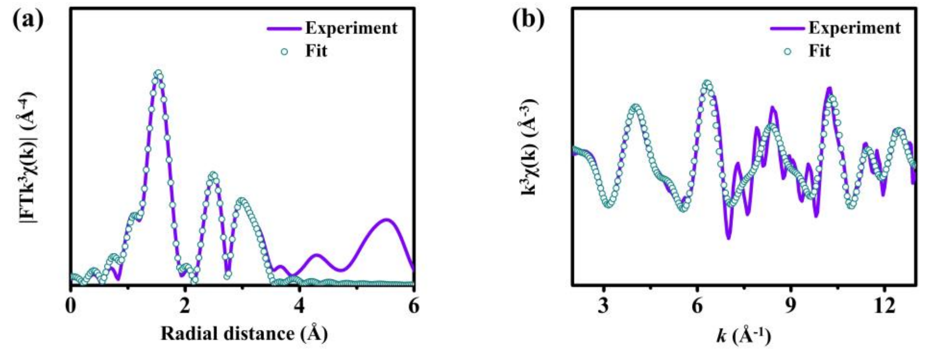

Supplementary Fig. 12. EXAFS spectra of CuO. Cu *K*-edge experimental and fitting Fourier transformed EXAFS spectra of CuO at (a) *R* space and (b) *K* space.

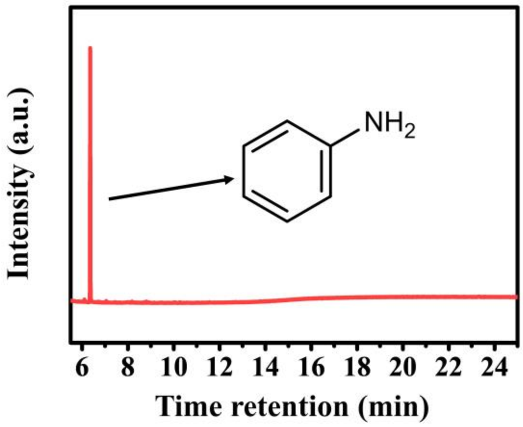

Supplementary Fig. 13. GC tests. GC result of Ph-NO_2 ERR for Cu_{SA}-Rh MAs/CF at -0.1 V (*vs.* RHE).

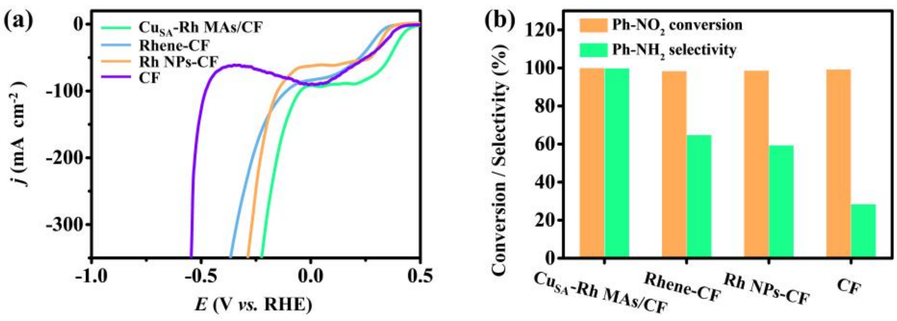

Supplementary Fig. 14. Electrocatalytic Ph-NO_2 ERR performance of various catalysts. (a) LSV curves of Cu_{SA}-Rh MAs/CF, Rhene-CF, Rh NPs-CF and CF in 1 M KOH + 5 mM Ph-NO_2 solutions. (b) Ph-NO_2 conversion and Ph-NH_2 selectivity of Cu_{SA}-Rh MAs/CF, Rhene-CF, Rh NPs-CF and CF at -0.1 V (*vs.* RHE).

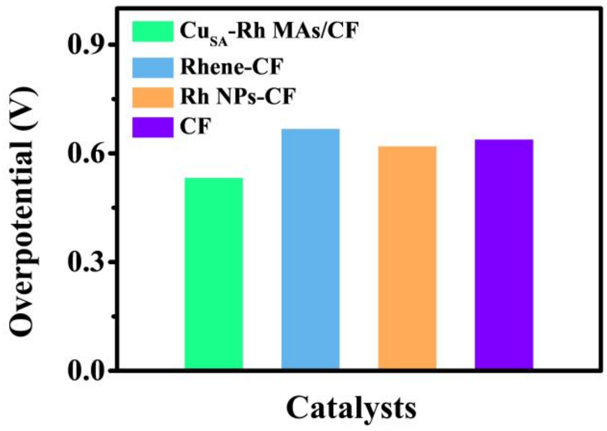

Supplementary Fig. 15. Overpotential comparison of various catalysts. Comparison of the overpotential for Cu$_{SA}$-Rh MAs/CF, Rhene-CF, Rh NPs-CF and CF relative to the standard potential of the Ph-NO_2 ERR at a current density of -50 mA cm^{-2}.

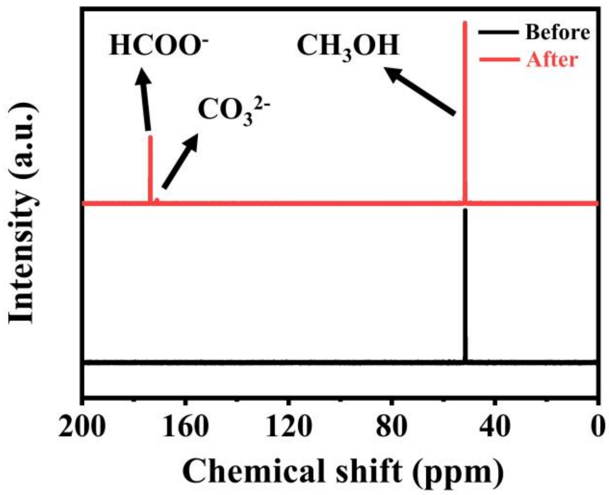

Supplementary Fig. 16. ^{13}C NMR spectra analysis. ^{13}C NMR spectra of products before and after 24 h MOR for Cu$_{SA}$-Rh MAs/CF at 1.4 V (*vs.* RHE).

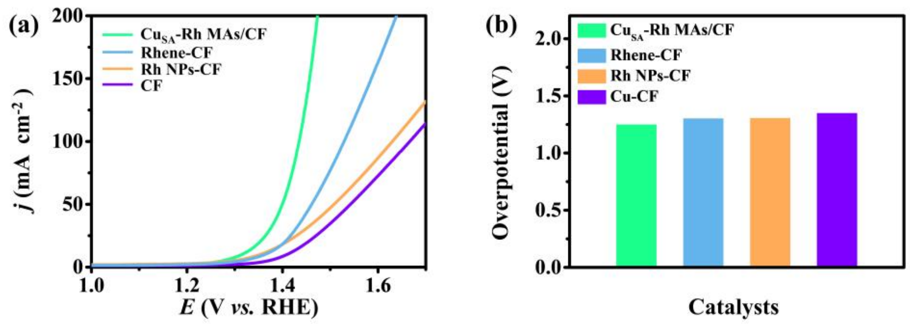

Supplementary Fig. 17. Electrocatalytic MOR performance of various catalysts. (a) LSV curves of Cu_{SA}-Rh MAs/CF, Rhene-CF, Rh NPs-CF and CF in 1 M KOH + 4 M CH_3OH solutions. (b) Comparison of the overpotential for Cu_{SA}-Rh MAs/CF, Rhene-CF, Rh NPs-CF and CF relative to the standard potential of the MOR at a current density of 20 mA cm^{-2}.

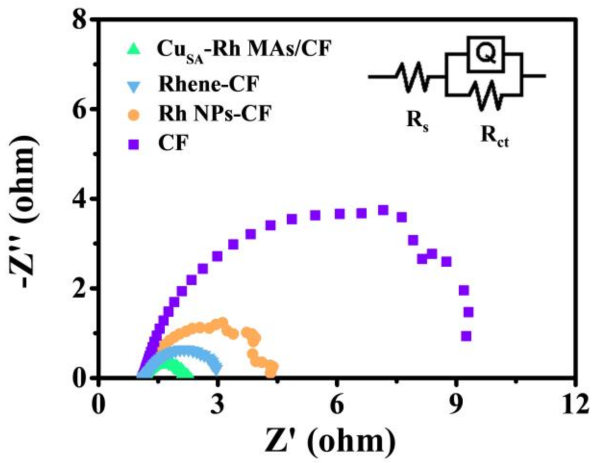

Supplementary Fig. 18. EIS tests. EIS plots of Cu_{SA}-Rh MAs/CF, Rhene-CF, Rh NPs-CF and CF in 1 M KOH solutions at applied potentials: -0.1 V (*vs*. RHE).

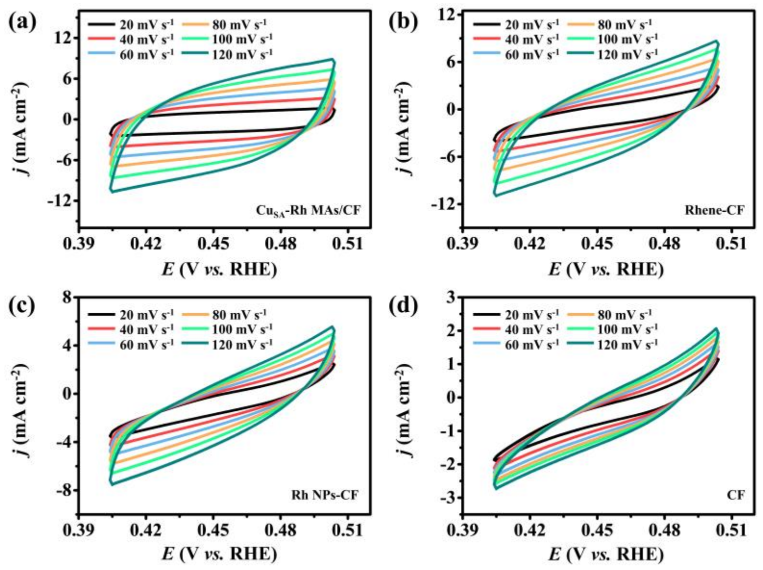

Supplementary Fig. 19. CV tests. CVs of (a) Cu_{SA}-Rh MAs/CF, (b) Rhene-CF, (c) Rh NPs-CF and (d) CF in the region of 0.404-0.504 V (*vs.* RHE).

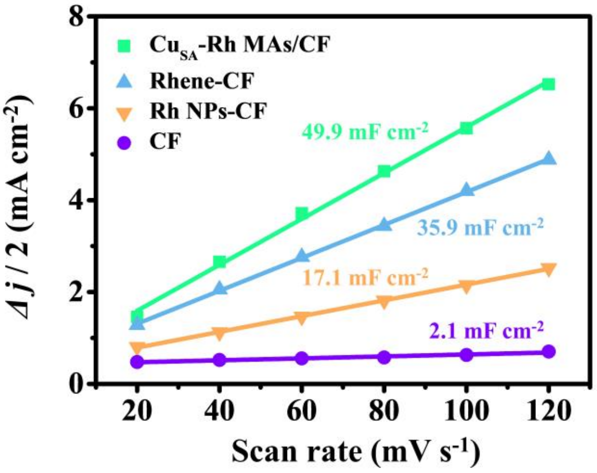

Supplementary Fig. 20. C_{dl} values. Capacitive current densities at 0.454 V (*vs.* RHE) derived from CVs against scan rates of Cu_{SA}-Rh MAs/CF, Rhene-CF, Rh NPs-CF and CF.

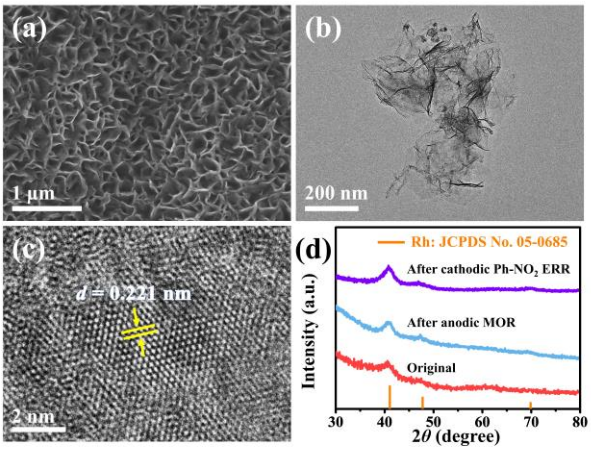

Supplementary Fig. 21. Morphological and structure characterization after stability testing. (a) SEM image of Cu_{SA}-Rh MAs/CF after stability testing. (b) TEM, and (c) HRTEM images of Cu_{SA}-Rh MAs after stability testing. (d) XRD patterns of Cu_{SA}-Rh MAs before and after stability testing on cathode and anode.

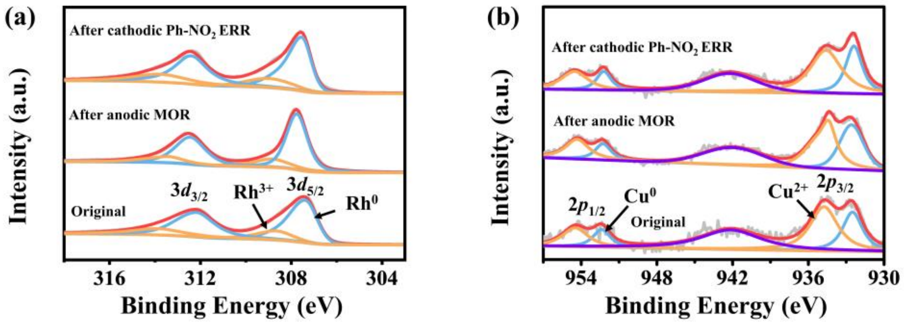

Supplementary Fig. 22. XPS results after stability testing. XPS spectra of (a) Rh 3*d* and (b) Cu 2*p* for Cu_{SA}-Rh MAs before and after stability testing on cathode and anode.

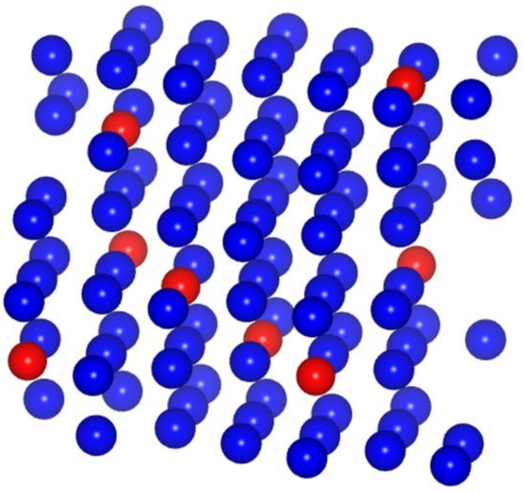

Supplementary Fig. 23. DFT calculation model. The optimized geometric structure model for Cu_{SA}-Rh MAs.

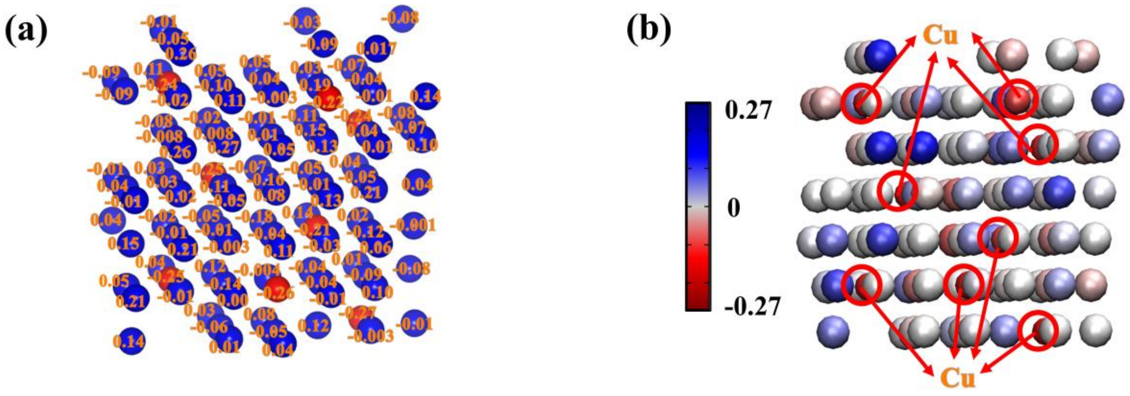

Supplementary Fig. 24. Bader charge calculation analysis. (a) Net charge obtained from bader charge analysis marked on Rh (blue balls) and Cu atoms (red balls). (b) Bader charge of Cu_{SA}-Rh (the balls marked by red circles represent the tendency for electron transfer on Cu single-atom and the unmarked balls represent the tendency for electron transfer on Rh host).

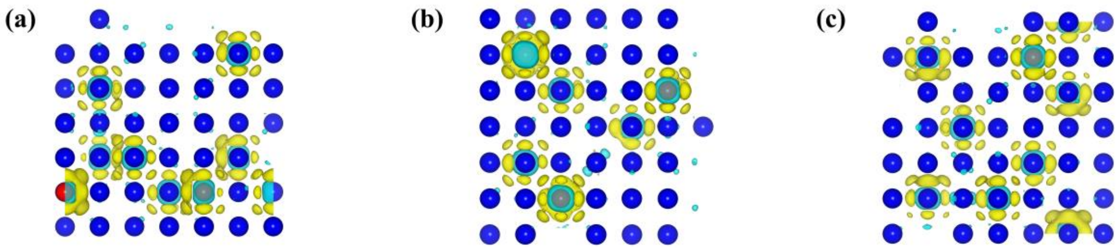

Supplementary Fig. 25. DFT calculation model. (a) Main view, (b) side view and (c) top view of charge density difference on Cu_{SA}-Rh. The blue and red spheres represent Rh and Cu atoms respectively, as well as the yellow and cyan indicate the charge depletion and accumulation areas.

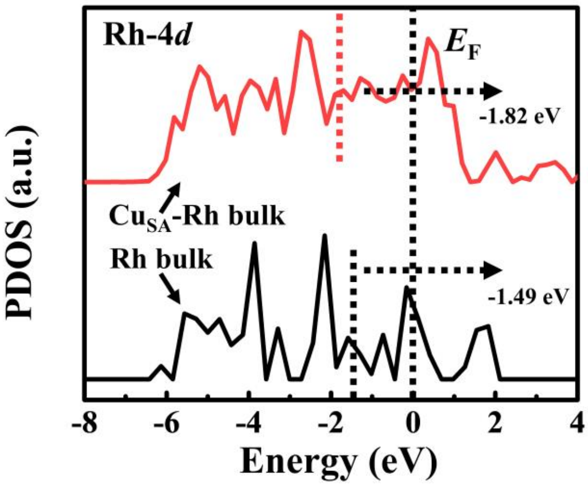

Supplementary Fig. 26. PDOS results. The PDOSs for Rh-4*d* of Rh bulk and Cu_{SA}-Rh bulk.

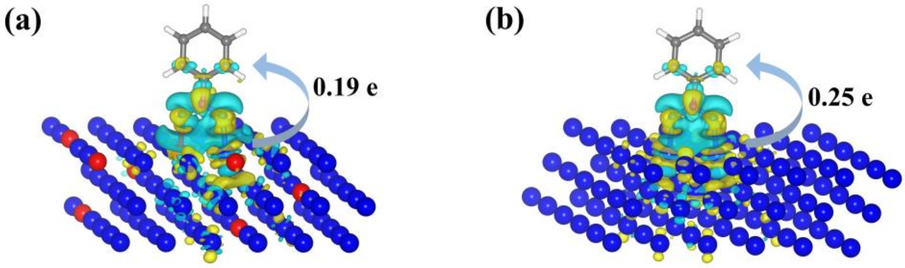

Supplementary Fig. 27. Charge density difference calculation analysis. Charge density difference for adsorbed Ph-NO_2* on (a) Cu_{SA}-Rh (111) and (b) Rh (111). The blue and red spheres represent Rh and Cu atoms respectively, as well as the yellow and cyan indicate the charge depletion and accumulation areas.

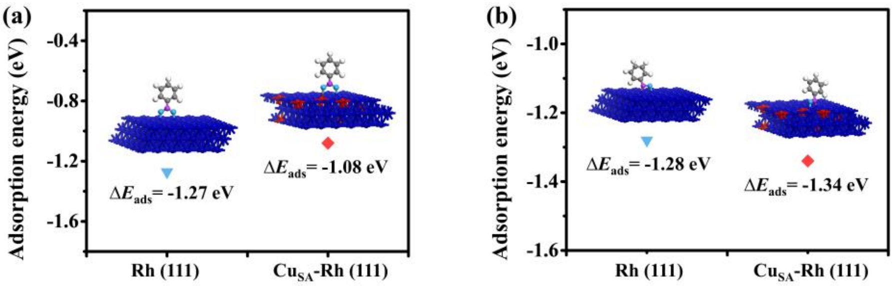

Supplementary Fig. 28. Adsorption energy calculation. Calculated adsorption energies of (a) Ph-NO_2* and (b) Ph-NO* on Rh (111) and Cu_{SA}-Rh (111).

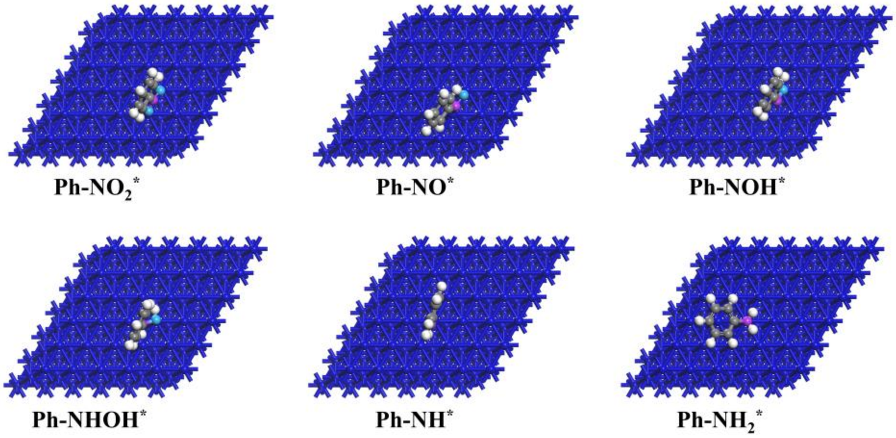

Supplementary Fig. 29. DFT calculation model. Optimized structures of Ph-NO_2 ERR intermediates on Rh (111). The blue, gray, cyan, purple, and white spheres represent Rh, C, O, N, and H atoms respectively.

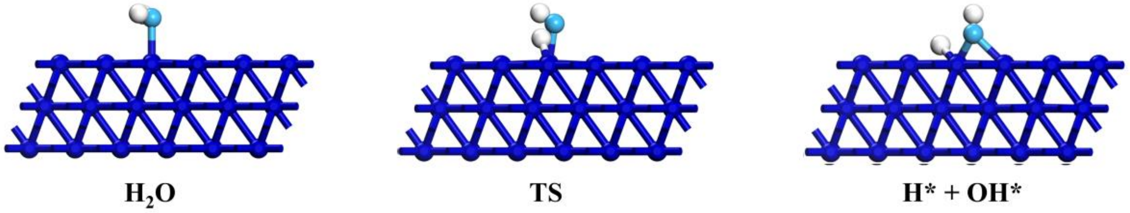

Supplementary Fig. 30. DFT calculation model. Optimized structures for the initial, transition and final states of H_2O dissociation on Rh (111). The blue, cyan, and white spheres represent Rh, O, and H atoms respectively.

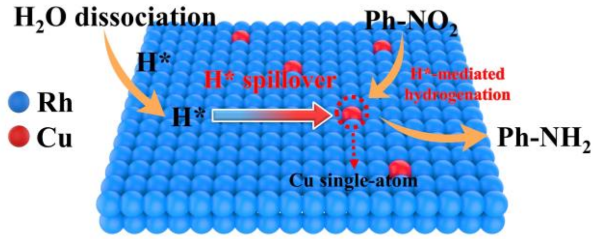

Supplementary Fig. 31. H*-spillover effect mechanism. Reaction mechanism diagram for hydrogen spillover effect on Cu_{SA}-Rh during Ph-NO_2 ERR process.

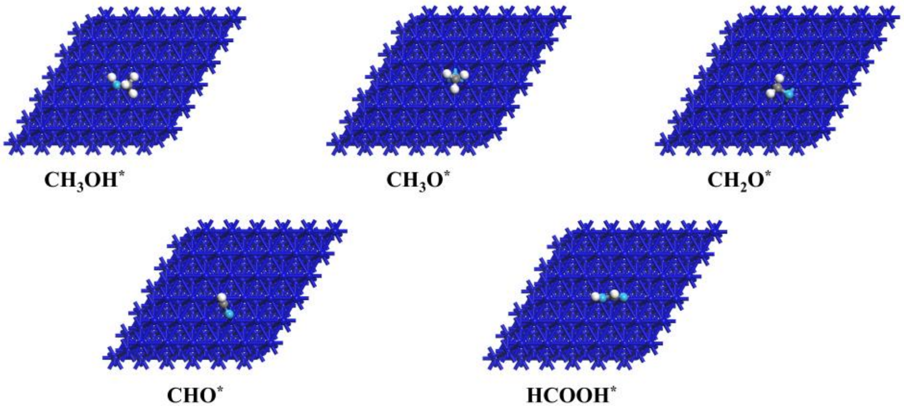

Supplementary Fig. 32. DFT calculation model. Optimized structures of MOR intermediates on Rh (111). The blue, gray, cyan, and white spheres represent Rh, C, O, and H atoms respectively.

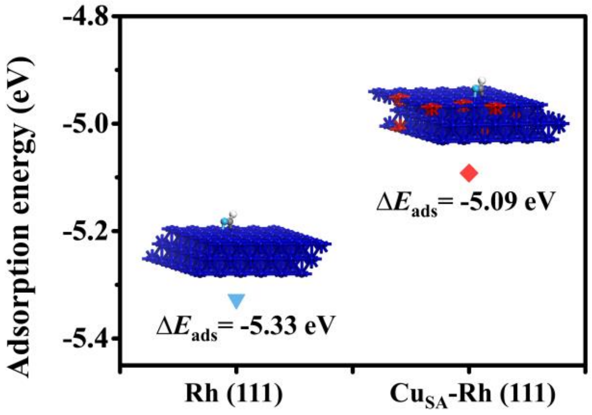

Supplementary Fig. 33. Adsorption energy calculation. Calculated adsorption energies of CHO* on Rh (111) and Cu_{SA}-Rh (111).

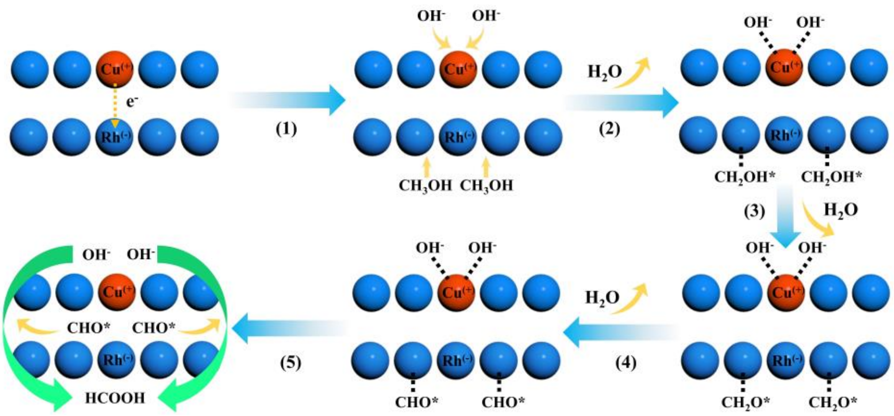

Supplementary Fig. 34. MOR mechanism. MOR mechanism diagram of synergistic catalysis induced by electronic effect on Cu_{SA}-Rh.

Supplementary Tables

Supplementary Table 1. EXAFS fitting parameters at the Cu *K*-edge for Cu_{SA}-Rh MAs.

Sample	Edge	Path	CN	R(Å)	σ^2(10^{-3} Å)	ΔE_0(eV)	R-factor
Cu_{SA}-Rh MAs	Cu-K	Cu-N	0.35	1.87	1.00	21.65	0.007
		Cu-O	1.27	2.04	9.63	19.69	
		Cu-Rh	4.78	2.63	6.32	7.39	

CN, coordination number; R, distance between absorber and backscatter atoms. σ^2, Debye-Waller factor; ΔE_0, the inner potential difference between the reference compound and the experimental sample. R-factor, the goodness of fit.

Supplementary Table 2. Bader charge analysis for Cu_{SA}-Rh. The electron transformation is 1.94e from Cu to Rh.

Atom species	Bader charge (e)
Rh	1.94
Cu	-1.94

Supplementary Table 3. Bader charge analysis for adsorbed Ph-NO_2* on Cu_{SA}-Rh (111) and Rh (111).

Species	Bader charge (e)
Cu_{SA}-Rh (111)	0.19
Rh (111)	0.25

Supplementary Table 4. Calculated adsorption energy values of Ph-NO_2* and Ph-NO* on Rh (111) and Cu_{SA}-Rh (111).

Reactant/Intermediates	Cu_{SA}-Rh (111)	Rh (111)
Ph-NO_2*	-1.08 eV	-1.27 eV
Ph-NO*	-1.34 eV	-1.28 eV

Supplementary Table 5. Calculated free energy values of various intermediates for Ph-NO_2 ERR pathway on Cu_{SA}-Rh (111) and Rh (111).

Reactant/Intermediates	Cu_{SA}-Rh (111)	Rh (111)
Ph-NO_2	0 eV	0 eV
Ph-NO_2*	-0.387 eV	-0.580 eV
Ph-NO*	-2.689 eV	-2.578 eV
Ph-NOH*	-2.462 eV	-2.212 eV
Ph-NHOH*	-1.725 eV	-1.915 eV
Ph-NH*	-4.555 eV	-4.095 eV
Ph-NH_2*	-3.923 eV	-5.072 eV
Ph-NH_2	-4.227 eV	-4.227 eV

Supplementary Table 6. Calculated energy values of the initial, transition and final states of H_2O dissociation on Cu_{SA}-Rh (111) and Rh (111).

Reactant/Intermediates	Cu_{SA}-Rh (111)	Rh (111)
H_2O (IS)	0 eV	0 eV
TS	0.832 eV	1.010 eV
H* + OH* (FS)	-0.237 eV	0.117 eV

Supplementary Table 7. Calculated H* adsorption energy values at different sites on Cu_{SA}-Rh (111).

Sites	Adsorption energy (eV)
A	-0.26
B	-0.27
C	-0.29
D	-0.31
E	-0.47

Supplementary Table 8. Calculated free energy values of various intermediates for MOR pathway on Cu_{SA}-Rh (111) and Rh (111).

Reactant/Intermediates	Cu_{SA}-Rh (111)	Rh (111)
CH_3OH	0	0
CH_3OH*	-0.400	-0.642
CH_3O*	-0.409	-0.492
CH_2O*	-0.068	-0.266
CHO*	0.614	-0.514
HCOOH*	0.217	0.110
HCOOH	0.647	0.647

Supplementary Table 9. Calculated adsorption energy values of CHO* on Rh (111) and Cu_{SA}-Rh (111).

Reactant/Intermediates	Cu_{SA}-Rh (111)	Rh (111)
CHO*	-5.09 eV	-5.33 eV

Supplementary References

1. Kresse G & Furthmüller J. Efficiency of ab-initio total energy calculations for metals and semiconductors using a plane-wave basis set. *Comput. Mater. Sci.* **6**, 15-50 (1996).
2. Blochl PE. Projector augmented-wave method. *Phys. Rev. B Condens. Matter* **50**, 17953-17979 (1994).
3. Perdew JP, et al. Atoms, molecules, solids, and surfaces: Applications of the generalized gradient approximation for exchange and correlation. *Phys. Rev. B Condens. Matter* **46**, 6671-6687 (1992).
4. Grimme S, Antony J, Ehrlich S & Krieg H. A consistent and accurate ab initio parametrization of density functional dispersion correction (DFT-D) for the 94 elements H-Pu. *J. Chem. Phys.* **132**, 154104 (2010).
